# Lineage- and stage-specific activity of antigen receptor gene enhancers during lymphocyte development

**DOI:** 10.3389/freae.2024.1489362

**Published:** 2024-12-16

**Authors:** Fei Ma, Fatima Zohra Braikia, Ranjan Sen

**Affiliations:** Gene Regulation Section, Laboratory of Molecular Biology and Immunology, National Institute on Aging, Baltimore, MD, United States

**Keywords:** enhancers, antigen receptor gene, lymphocyte development, lineage specific, stage specific, transcription factors (TF)

## Abstract

Lymphocyte development culminates with generation of mature B and T cells that express unique antigen receptors on the cell surface. Genes that encode the two chains of B or T cell receptors are generated via DNA recombination and expressed sequentially during development, guided by locus activating enhancer sequences. In this review we summarize our understanding of molecular mechanisms that activate these enhancers in a lineage and developmental stage-specific manner. We draw attention to 1) the distinction between chromatin accessibility and transcriptional activation of these loci, 2) incomplete understanding of mechanisms that regulate B versus T cell-specific enhancer activity and 3) transcription factors that contribute to stage-specific enhancer activation within each lineage.

## Introduction

B and T lymphocytes of the adaptive immune system protect against a variety of pathogens via unique receptors expressed on the cell surface. The wide diversity of such antigen receptor specificities ensures high likelihood of recognizing newly emerging, or previously encountered, pathogens. Antibodies, that constitute B cell antigen receptors (BCRs) are heterotetramers of two identical heavy chains (IgH) and two identical light chains (IgL) of either kappa (Igκ) or lambda (Igλ) type. T cell receptors (TCRs) confer antigen specificity to T lymphocytes via heterodimers consisting of alpha (TCRα) and beta (TCRβ) chains or gamma (TCRγ) and delta (TCRδ) chains. The unique recognition specificity of each lymphocyte is determined by variable N-terminal domains in BCRs and TCRs. These domains, and thereby receptor diversity, are generated during lymphocyte development.

## Antigen receptor genes are assembled by V(D)J recombination

Unlike all other mammalian genes, loci that encode antigen receptors are composed of gene segments rather than fully functional genes. The variety, and thereby diversity, of antigen receptors is generated in part by randomly assorting hundreds of gene segments during lymphocyte development (Figure 1A). The mouse *Igh* locus, for example, contains several hundred variable (V_H_) gene segments (C57BL/6 mice have 110 functional V_H_ genes and 85 V_H_ pseudogenes), 8–12 diversity (D_H_) gene segments and 4 joining (J_H_) gene segments distributed over 3 Mb (Johnston et al., 2006) (Figure 1B). The N-terminal variable domain of antibody heavy chains is assembled by genomic juxtaposition of one V_H_, one D_H_ and one J_H_ gene segment by a process known as V(D)J recombination (Chowdhury and Sen, 2004; Jung and Alt, 2004; Jung et al., 2006; Kumari and Sen, 2015; Proudhon et al., 2015). Variable domains of TCRβ and TCRδ chains are also assembled by recombining three gene segments. By contrast, Ig light chain (Igκ or Igλ) and TCRα and TCRγ chain genes require only one recombination event between a variable and a joining gene segment to generate functional genes (Krangel, 2009; Collins and Watson, 2018). These gene rearrangements are mediated by the identical nuclear enzymatic machinery in both lineages. Key amongst these are the recombination-activating gene products, RAG1 and RAG2, that introduce double-strand DNA breaks to initiate the process and are expressed together only in developing lymphocytes (Schatz et al., 1989; Oettinger et al., 1990; Teng and Schatz, 2015; Lescale and Deriano, 2017; Lin et al., 2018; Christie et al., 2022). Thereafter, ubiquitously expressed proteins of the non-homologous end joining pathway are recruited to complete the process (Lieber, 2010; Wang et al., 2020; Zhao et al., 2020).

**FIGURE 1:**
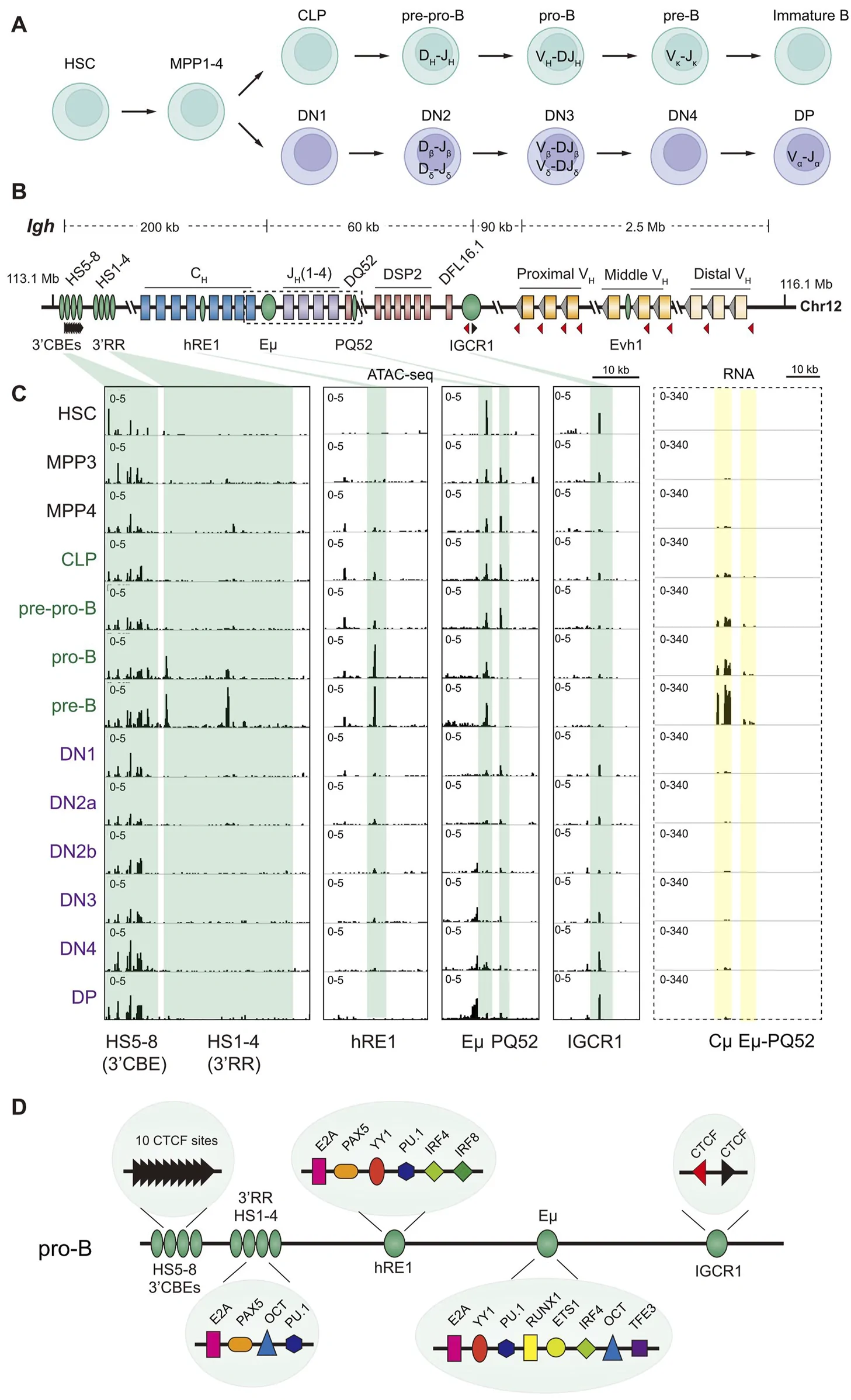
*Igh* locus regulatory sequences and transcription during B cell development. **(A)** Overview of adult hematopoiesis in mice. Long term reconstituting hematopoietic stem cells (HSC) differentiate via multipotential progenitors (MPP1-4) to B lymphocytes in the bone marrow (top row) and T lymphocytes (bottom row) in the thymus. Common lymphoid progenitors (CLP), though multipotent, are largely B cell precursors that differentiate via several intermediate stages to immunoglobulin (Ig) expressing mature B cells in the bone marrow. Multipotency is lost around the pre-pro-B cell stage where immunoglobulin heavy chain (*Igh*) gene rearrangements initiate. Ig light chain rearrangements occur in the pre-B cells. Multipotent cells that migrate to the thymus (DN1, a heterogenous cell subset) commit to T lineage differentiation in DN2 cells where T cell receptor β (*Tcrb*) gene rearrangements initiate. *Tcra* rearrangements occur in DP cells. **(B)** Schematic representation of the 3 Mb mouse *Igh* locus located on chromosome 12 (coordinates shown are mm10). Variable (V_H_), diversity (DQ52, DSP2 and DFL16.1), and joining (J_H_) gene segments are shown as yellow, pink, and purple boxes. Blue boxes represent constant region (C_H_) exons. Grey triangles adjacent to the V_H_ gene segments indicate recombinase signal sequences (RSS) required for gene rearrangements. RSS adjacent to diversity and joining segments are not shown. Cis-regulatory sequences discussed in this review are shown as green ovals, including enhancers (Evh1, Eµ, hRE1, and 3′RR), intergenic CTCF site IGCR1 and 3′CBEs. CTCF binding sites and orientation are shown by black and red triangles. The region highlighted by a dashed box is expanded in the right panel of part C to display RNA-seq data. **(C)** ATAC-seq (left panel) and RNA-seq (right panel) profiles of the 3′ part of the *Igh* locus during hematopoiesis obtained from the Immunological Genome project (Yoshida et al., 2019). ATAC-seq patterns covering known cis regulatory sequences are shown as identified below the tracks. For RNA-seq the pattern of the locus in the dashed box in part B is shown. **(D)** Transcription factors that binds to previously identified regulatory sequences in the 3′ *Igh* domain in pro-B cells are shown. The summary combines *in vitro* protein binding studies and *in vivo* analysis by chromatin immunoprecipitation assays (ChIP) (Kumari et al., 2018; Medvedovic et al., 2013; Kleiman et al., 2016; Ernst and Smale, 1995; Henderson and Calame, 1998; Lin et al., 2010; Khamlichi et al., 2000; Loguercio et al., 2018), and does not represent the numbers of each protein binding site.

Despite a shared rearrangement mechanism, BCR genes recombine fully only in the B cells and TCR genes rearrange only in T cells. This lineage specificity has been understood in term of the accessibility hypothesis (Yancopoulos and Alt, 1985; Stanhope-Baker et al., 1996; Krangel, 2003). Namely, RAG proteins can access and act upon BCR loci, but not TCR loci, in B cell precursors and, conversely, only TCR loci (but not BCR loci) in precursor T cells. Additionally, antigen receptor gene recombination is developmentally segregated within each lineage. During B cell development, rearrangement and expression of *Igh* genes occurs first at the pro-B cell stage, followed by *Igk* and *Igl* genes in pre-B cells (Figure 1A) (Lescale, 2016; Borghesi et al., 2004; Hardy et al., 1991). In the T cell lineage, *Tcrb* rearrangements and expression in CD4^−^CD8^−^ (double negative, DN) cells precede *Tcra* rearrangements which occur at the later CD4^+^CD8^+^ (double positive, DP) stage (Figure 1A) (Krangel, 2009; Christie et al., 2022; Rothenberg and Taghon, 2005). The lineage-specific accessibility hypothesis can be extended to account for stage-specificity of rearrangements within each lineage. For this, the idea is that *Igh* loci rearrange in pro-B cells when RAG proteins do not have access to *Igk* or *Igl* loci. The latter become accessible only at the pre-B cell stage once *Igh* rearrangements are completed. Similarly, only the *Tcrb* locus, but not *Tcra* locus, is accessible to RAG proteins in DN cells, the latter becoming accessible to RAGs at the subsequent DP stage. *Igl*, *Tcrg* and *Tcrd* loci are similarly regulated but will not be discussed in detail in this review due to space constraints.

## Enhancers regulate antigen receptor gene assembly and expression

Enhancers were identified as regulatory sequences that activated transcription in a position (5′ or 3′ of a gene promoter) and orientation (relative to gene transcription) independent manner (Banerji et al., 1981). They function by recruiting DNA binding proteins to specific regions of the genome. These proteins (also referred to as transcription factors), in turn, recruit accessory proteins that result in the formation of multi-protein complexes on enhancers (Zabidi and Stark, 2016; Haberle and Stark, 2018; Jindal and Farley, 2021; Panigrahi and O'Malley, 2021). The numbers and layers of accessory proteins recruited likely varies between enhancers and has not been fully described for any enhancer. One of the best-known accessory proteins is the CREB binding protein (CBP) and its closely related family member, p300. CBP/p300 are histone acetyl transferases (HATs) that acetylate lysine 27 on histone H3 leading to the epigenetic modification H3K27ac (Heintzman et al., 2007; Creyghton et al., 2010; Rada-Iglesias et al., 2011; Calo and Wysocka, 2013). This mark is associated with gene transcription and active enhancers, and CBP/p300 are also referred to as co-activators (Weinert et al., 2018; Narita et al., 2021). Active enhancers are also associated with high chromatin accessibility as measured by sensitivity to endonucleases. The most recent iteration of this is the assay for transposase accessible chromatin followed by sequencing (ATAC-seq) that closely aligns with DNase I hypersensitivity assays (Buenrostro et al., 2015). Enhancer sequences that lack H3K27ac but are marked by H3K4me1 have been referred to as poised enhancers that are ready for activation (Creyghton et al., 2010; Lesch and Page, 2014; Crispatzu et al., 2021; Jenuwein and Allis, 2001; Klemm et al., 2019). DNA-bound transcription factors may also recruit co-repressor complexes, such as NcoR/SMRT and mSin3 that contain histone deacetylases, that are associated with gene repression (Adams et al., 2018; Watson et al., 2012; Wong et al., 2014). Because co-activators and co-repressors are expressed in most cell types, their tissue-specific utilization likely resides in the spectrum of transcription factors recruited to tissue-specific enhancers. Enhancer sequences have been identified in murine and human antigen receptors gene loci (Rodriguez-Caparros et al., 2020; Kasprzyk et al., 2021). Most of the analyses have been carried out with the murine enhancers, which bear all hallmarks of traditional transcriptional enhancers, that are the focus of this review.

Several observations substantiate the role of enhancers in determining lineage and developmental timing of antigen receptor gene activation. First, deletion of enhancers associated with these loci demonstrates that they are necessary for developmentally appropriate activation of each locus (further discussed below). Second, genetic substitution of enhancers partially recapitulates regulatory features of the locus from which it is derived. For example, replacement of an enhancer in the *Tcrb* locus (Eβ) by one from *Igh* (Eµ) induces *Tcrb* transcription in B lymphocytes (Bories et al., 1996). In another study, substitution of Eβ by an enhancer associated with the later activated *Tcra* locus reduced *Tcrb* transcription in early stage DN cells but activated transcription in DP cells where *Tcra* genes rearrange (Senoo et al., 2001). Similarly, substitution of the iEκ enhancer associated with the *Igk* locus that is activated later in B cell development with Eµ led to premature *Igk* transcription and rearrangements in pro-B cells (Inlay et al., 2006). Third, antigen receptor gene enhancers direct lineage and developmentally stage-specific activation of transgenes in mice. Eμ, for example, is necessary and sufficient to activate *Ig* or heterologous genes in B cells of transgenic mice (Adams et al., 1985). Similarly, Eβ, Eα and Eµ have been shown to activate transcription and V(D)J recombination in transgenic mini loci (Capone et al., 1993; Okada et al., 1994; Ferrier et al., 1990). Eβ has also been used to activate other transgenes in T cells. In a notable exception, Eµ activates transgenes in both B and T cell lineages (Ferrier et al., 1990). Eμ promiscuity is also reflected in D_H_ to J_H_ rearrangements and transcription of the *Igh* locus in a large proportion of thymocytes in wild type mice (Kurosawa et al., 1981; Born et al., 1988; Allman et al., 2003; Kumari et al., 2018). These transgenic experiments show that antigen receptor gene enhancers are sufficient to activate transgenes integrated at different genomic locations, reminiscent of the properties of locus control regions (Jenuwein et al., 1993). Thus, a few hundred nucleotides constituting these enhancers carry the information content that specifies tissue-specific gene activation. The sections below address mechanisms by which such specificity is achieved.

## Enhancers that regulate immunoglobulin gene rearrangements

### Enhancer control of Igh expression

B cells develop from hematopoietic stem cells (HSC) through intermediates that retain various levels of multipotentiality. Commitment to differentiation into B cells occurs close to the pre-pro-B cell differentiation stage (Figure 1A). *Igh* rearrangements initiate in these cells with D_H_ rearrangements, followed by V_H_ rearrangements at the pro-B cells stage (Alt et al., 1984). IgH expression is a checkpoint during B cell development. Only IgH-expressing pro-B cells differentiate to pre-B cells where Ig light chain genes (*Igk* and *Igl*) rearrange. Expression of light chain permits immature B cells to express membrane antibody molecules of the IgM isotype.

The 3′ *Igh* domain, extending from the intergenic control region 1 (IGCR1) to 3′ CTCF binding elements (3′CBE), within which the first rearrangements occur is marked by several regions of high chromatin accessibility in pro-B cells (Figure 1C, left panel). At the 5′ boundary two CTCF binding sites within IGCR1 regulate *Igh* rearrangements. Their mutation or deletion accentuates use of the closest V_H_ gene segments thereby severely compromising *Igh* diversity, as well as permits V_H_ rearrangements to unrearranged D_H_ gene segments (Featherstone et al., 2010; Guo et al., 2011; Lin et al., 2015; Qiu et al., 2018; Giallourakis et al., 2010). The intronic enhancer, Eµ, and a promoter (PQ52) associated with the 3′-most D_H_ gene segment, DQ52, are marked by two closely associated ATAC-sensitive regions. In pro-B cells Eµ regulates histone modifications in the 3′ *Igh* domain (Chakraborty et al., 2009), induces transcription of the unrearranged (germline) locus and activates rearrangements. Deletion of Eµ reduces D_H_ recombination substantially (∼80%) and virtually abolishes V_H_ recombination (Chakraborty et al., 2009; Perlot et al., 2005; Afshar et al., 2006; Bolland et al., 2007).

Several additional chromatin accessible sites are evident moving 3′ from Eµ (Figure 1C, left panel). hRE1 is an enhancer located between Cγ1 and Cγ2b IgH isotypes (Medvedovic et al., 2013; Predeus et al., 2014). hRE1 is not required for *Igh* rearrangements in pro-B cells but promotes class switch recombination (CSR) to IgG3, IgG2b and IgG2a isotypes during immune responses (Amoretti-Villa et al., 2019). The 3′ regulatory region (3′RR), located 3′ of the last Cα exons, consists of a cluster of four B cell-specific transcriptional enhancers that span 28 kb (Lieberson et al., 1991; Giannini et al., 1993; Matthias and Baltimore, 1993; Madisen and Groudine, 1994; Michaelson et al., 1995). Like hRE1, the 3′RR contributes primarily to CSR, but not to control of *Igh* rearrangements in pro-B cells (Vincent-Fabert et al., 2010; Rouaud et al., 2012; Saintamand et al., 2015; Bruzeau et al., 2021; Oudinet et al., 2020). Finally, 3′CBE is a cluster of CTCF binding chromatin accessible regions that mark the 3′ boundary of the *Igh* topologically associated domain (TAD) (Garrett et al., 2005; Vian et al., 2018). Accordingly, its deletion leads to transcriptional activation of genes located further 3′ that are not normally expressed in pro-B cells (Volpi et al., 2012; Zhang et al., 2021). Recently, an additional enhancer has been identified within the V_H_ genes. Its deletion affects recombination of closely positioned V_H_ gene segments (Bhat et al., 2023). Thus, Eµ constitutes the only validated regulatory sequence associated with both *Igh* locus activation and rearrangements in pre-pro- and pro-B cells.

Though originally identified as a transcriptional enhancer and proposed to be important for transcription of rearranged *Igh* alleles, several lines of evidence suggest that Eµ is not required for *Igh* expression in mature B cells or during immune responses. Eckhardt and colleagues first demonstrated that VDJ ‘knock in’ *Igh* alleles that lacked Eµ permitted normal B cell development and function (Li and Eckhardt, 2009; Li et al., 2010). Other studies in germline Eµ-deficient mice indicate that immune responses are not affected substantially despite smaller numbers of mature B cells in these strains (Perlot et al., 2005; Marquet et al., 2014). However, Eμ is essential for B cell-specific transcription of functionally rearranged *Igh* transgenes in mice. Eμ has also been shown to be a diversity activating sequence (DIVAC) that promotes activation-induced deaminase dependent somatic hypermutation of Ig sequences, a process that occurs only during peripheral immune responses (Buerstedde et al., 2014). We believe that additional studies of the role of Eμ in mature B cells are warranted.

### Mechanisms of Eµ activation

Eµ binds many transcription factors (Figure 1D) (Kumari et al., 2018; Kleiman et al., 2016; Ernst and Smale, 1995; Henderson and Calame, 1998; Lin et al., 2010). However, none of these easily explain lineage- or developmental stage-specificity of *Igh* activation. Most of these proteins are widely expressed in hematopoietic cells, such YY1, RUNX family members, ETS proteins, OCT proteins and bHLH-zip proteins. Others, like PU.1 and E2A have more restricted tissue distribution. PU.1 is expressed at highest levels in myeloid cells where it has been proposed to act as a pioneer factor (Heinz et al., 2010). Lower levels of PU.1 present at early hematopoietic stages (such as HSC, CMP and CLP) are maintained throughout B cell development but extinguished during T cell differentiation (Heinz et al., 2010; Iwasaki et al., 2005; Dakic et al., 2007; Pang et al., 2018). E2A is also expressed in HSC through CLP stages but further up-regulated in pre-pro-B cells and thereafter (Semerad et al., 2009; Fischer et al., 2020; Aubrey et al., 2022). Based on early transfection experiments it was proposed that Eµ function is generated by combinatorial activity of different Eµ-binding proteins, especially combinations of ETS proteins and E2A (Ernst and Smale, 1995; Nelsen and Sen, 1992; Nelsen et al., 1993). Which combinations are most important in the endogenous context have not been identified.

Eμ function is also modulated by 5′ and 3′ flanking matrix attachment regions (MARs) (Scheuermann and Garrard, 1999). These are A/T-rich sequences that bind several transcription factors, including Cux/CDP, Satb1 and Bright (Romig et al., 1992; Weitzel et al., 1997; Dickinson et al., 1992; Herrscher et al., 1995; Alvarez et al., 2000; Dobreva et al., 2003). In transgenic studies MAR sequences are essential to fully reveal Eμ activity as reflected in transcription activation, especially at a distance, and extension of chromatin accessibility (Jenuwein et al., 1997; Forrester et al., 1999; Fernández et al., 2001; Forrester et al., 1994). These effects of MARS are independent of transcription factor binding to Eμ, suggesting that factor binding is not sufficient for Eμ function (Fernández et al., 2001). Unlike the effects of deleting Eμ, however, deletion of one or both *Igh* MARs from the endogenous locus does not affect V(D)J recombination regulated by Eμ or B cell development (Sakai et al., 1999). The role of MARs and their relationship to enhancer function awaits further studies.

While Eµ is both necessary and sufficient for gene activation, examination of chromatin accessibility throughout hematopoiesis reveals hitherto unstudied complexities. First, accessibility at closely positioned Eµ and DQ52 promoter is evident in HSC and maintained throughout developmental stages that precede B lineage commitment (Figure 1C, left panel). Absence of the DQ52 ATAC peak in pro-B cells likely reflects loss of that region by D_H_ and V_H_ recombination. The most parsimonious explanation is that Eµ is not B lineage specific though it drives transgenic expression largely in B and T lymphocytes. Alternatively, it is possible that though Eµ is accessible at earlier stages, it does not have enhancer activity until pre-pro- or pro-B cell stages. This is reminiscent of poised enhancers that are chromatin accessible but lack H3K27ac. Consistent with the latter hypothesis, transcription (Figure 1C, right panel) and H3K27ac modifications (not shown) are higher in pro-B cells compared to HSC (Choukrallah et al., 2015). We surmise that binding of hematopoietic transcription factors to Eµ marks this site for later activation in B lineage cells. Comprehensive analyses of enhancer function throughout hematopoiesis will be required to understand the underlying mechanisms.

Lack of simple concordance between ATAC sensitivity and enhancer function raises two questions. First, what transforms a chromatin accessible, but transcriptionally silent, enhancer into a functionally active enhancer in the B lineage? Our working hypothesis is that chromatin accessibility throughout hematopoiesis marks a poised enhancer that is transcriptionally activated in pre-pro- and pro-B cells. How many transcription factor binding sites are required to poise the enhancer for B lineage-specific activation and which factors convert a pre-marked but inactive enhancer to an active one remain to be discovered. Second, why does Eµ bind so many different transcription factors? One possibility is that some of these factors may suppress enhancer activity in other hematopoietic lineages. The most closely related one is developing T cells where *Tcr* genes recombine. Eµ chromatin accessibility and associated transcription is considerably lower in T lineage precursors (DN1-DP stages) compared to pro-B cells (Figure 1C, left panel), though they express many of the same transcription factors. This reduced accessibility drives low levels of *Igh* D_H_ rearrangements in DP cells. We hypothesize that sub-optimal activation of Eµ in T cell precursors precludes compete V(D)J recombination and, thereby, the possibility of co-expressing functional IgH and TCRα/β chains in the same cell.

### Enhancer control of Igk expression

Surface Ig heavy chain expression in pro-B cells triggers a proliferative burst that culminates with production of pre-B cells (Figure 1A). The bulk of Ig light chain gene rearrangements take place in these cells. In this review we focus on the *Igk* locus that encodes more than 90% of light chain protein in mice. The mouse *Igk* locus spans 3.2 Mb, most of which encodes 96 functional Vκ gene segments (Figure 2A). Clustered at the 3′ end are 4-5 Jκ gene segments, one exon encoding the constant region of Igκ (Cκ) and several regulatory sequences (Figure 2A). One recombination event creates VκJκ junctions that encode Igκ.

**FIGURE 2:**
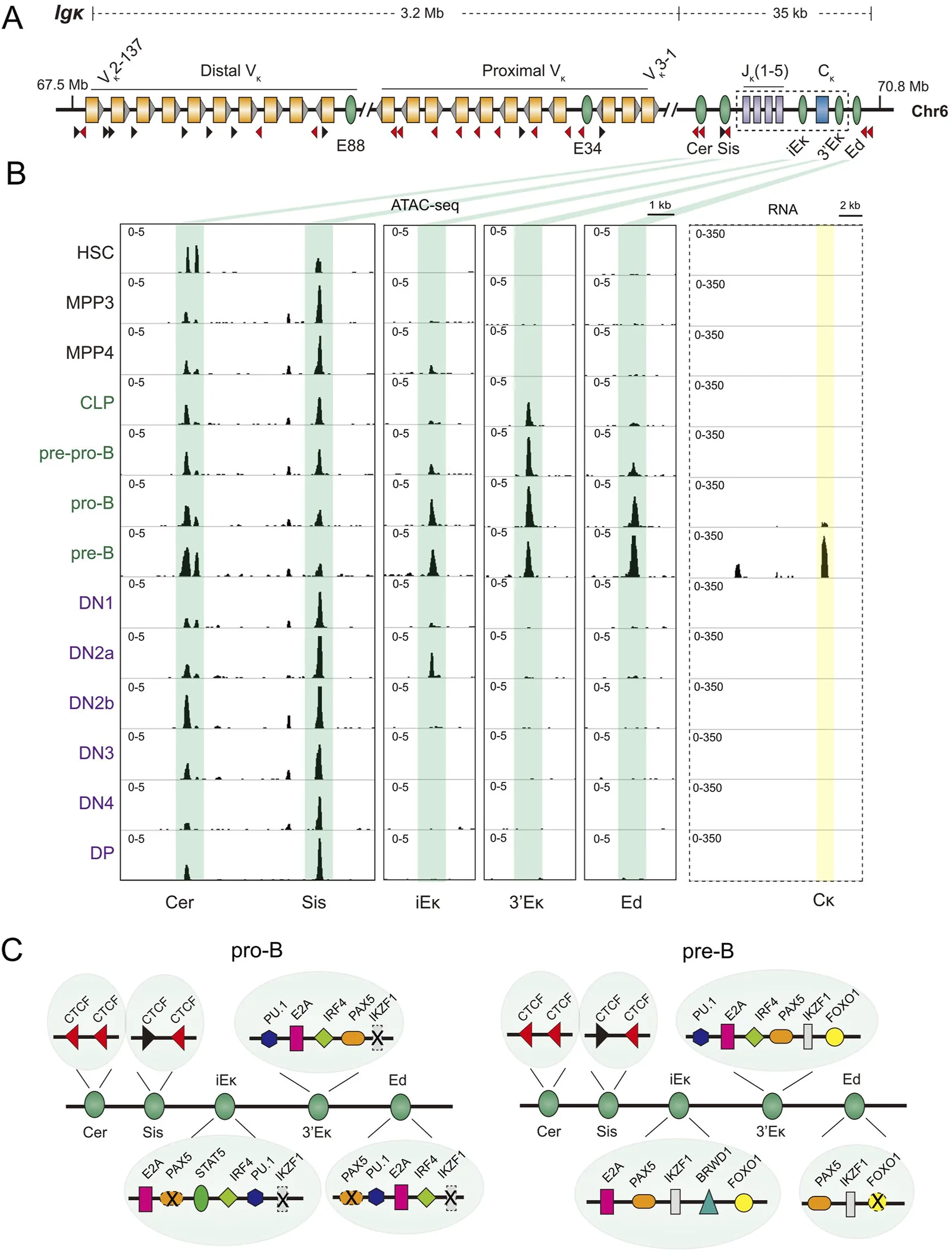
*Igk* locus regulatory sequences and transcription during B cell development. **(A)** Schematic representation of the mouse immunoglobulin kappa chain (*Igk*) locus, spanning approximately 3 Mb on chromosome 6 (coordinates are in mm10). Variable (Vκ) and joining (Jκ), segments, and the Cκ exon are depicted as yellow, purple, and blue boxes, respectively. Grey triangles adjacent to the Vκ gene segments indicate RSSs. Cis-regulatory elements, including enhancers (E88, E34, iEκ, 3′Eκ, Ed), contracting element for recombination (Cer), and silencer in the intervening sequence (Sis), are shown as green ovals. CTCF binding sites and orientations are shown by black and red triangles. The 3′ region of the locus, highlighted by a dashed box, is enlarged in part B to display RNA-seq data. **(B)** ATAC-seq (left panel) and RNA-seq (right panel) profiles of the 3′ region of the *Igk* locus during hematopoiesis obtained from the Immunological Genome project (Yoshida et al., 2019). ATAC-seq patterns covering known cis regulatory sequences are shown as identified below the tracks. For RNA-seq, the pattern of the locus in the dashed box in part **(A)** is shown. **(C)** Transcription factors that bind to previously identified regulatory sequences in the 3′ *Igk* domain in pro-B and pre-B cells are shown. The summary combines *in vitro* protein binding studies and *in vivo* analysis by chromatin immunoprecipitation (ChIP) (Lin et al., 2010; Mandal et al., 2011; Mandal et al., 2015; Shaffer et al., 1997; Lu et al., 2003; Ochiai et al., 2012; Revilla-I-Domingo et al., 2012; Schwickert et al., 2014; Stadhouders et al., 2014; Ferreiros-Vidal et al., 2013; Loguercio et al., 2018), and does not represent the numbers of each protein binding site. Sites depicted with dashed lines and a cross identify unoccupied sites at the specified developmental stage (Ribeiro de Almeida et al., 2015).

Multiple enhancers control *Igk* recombination. The intronic enhancer, iEκ, appears most important. iEk is marked by repressive H3K27me3 modification in pro-B cells that is replaced by acetylated histone 4 in pre-B cells where *Igk* genes recombine (Inlay et al., 2006). In its absence Vκ recombination is reduced about 10-fold (Xu et al., 1996). Residual recombination is lost upon additionally deleting 3′Eκ (Inlay et al., 2002), though deletion of 3′Eκ by itself has little/no effect (Inlay et al., 2002). A third enhancer, Ed, located further 3′ also has little effect when deleted alone, but the double deletion of Ed with 3′Eκ abolishes *Igk* rearrangements (Zhou et al., 2010). Thus, iEκ is insufficient for recombination, whereas 3′Eκ plus Ed together are relatively weak activators of *Igk* rearrangements. 3′Eκ and Ed may function as shadow enhancers alongside an active iEκ (Hobert, 2010). Loss of 3′Ek is accompanied by substantially reduced H3 acetylation at the Jκ gene segments in pre-B cells. Ed deletion has a smaller effect, but loss of both 3′Ek and Ed abolishes H3ac at Jκs (Zhou et al., 2010). Though histone acetylation status of iEκ deleted alleles has not been characterized, these results cumulatively suggest that enhancer activation reflected in active histone modifications correlates with induction of rearrangements. To what extent such modifications contribute to V(D)J recombination *per se* remains to be determined. Moreover, because different measures of locus activation have been used (many of which preceded the chromatin immunoprecipitation (ChIP) era), systematic studies of WT and mutated loci are needed to understand molecular connections between enhancers, chromatin states, transcriptional activation and recombination.

In addition to the classical enhancers, two elements, the contracting element for recombination (Cer) and silencer in the intervening sequence (Sis), regulate *Igk* rearrangements (Xiang et al., 2011; Xiang et al., 2013). Deletion of these elements skews the rearranged repertoire of Vκ genes to those located closer to the 3′ end of the locus (proximal Vκ genes). Specifically, Vκ genes lying within 100 kb of Jκs account for 25% of the repertoire in the absence of Sis and 62% of the repertoire in the absence of Cer, compared to 10% in the wild type context (Xiang et al., 2011; Xiang et al., 2013). The effects are even more pronounced when both elements are missing (Xiang et al., 2014). Their activity is likely conferred by oriented CTCF binding sites in each element, much like IGCR1 in *Igh*. However, the role of CTCF has not been directly confirmed by point mutations of these sites. Like *Igh*, additional enhancers within the Vκ region promote recombination of nearby gene segments (Barajas-Mora et al., 2019; Barajas-Mora et al., 2023). The greater effects of enhancers located near the joining gene segments (J_H_ and Jκ) at both immunoglobulin loci for gene rearrangements may be via their role in establishing RAG1/2-rich recombination centers at which antigen receptor gene rearrangements are initiated.

### Mechanisms of Igk activation

To what extent can developmental timing of *Igk* rearrangements be explained by enhancer activation by transcription factors? iEκ has been best studied in this regard. Early studies showed that mutating the NF-κB binding site in iEκ had little effect on rearrangements, whereas mutating two ‘E’ elements significantly reduced iEκ function (Inlay et al., 2004). These E elements bind E2A and bHLH-zip proteins such as TFE3 *in vitro* (Staudt and Lenardo, 1991). However, the importance of these motifs for iEκ function does not provide a ready explanation for developmental timing of *Igk* activation, in part because similar motifs are also found in Eμ that is activated at an earlier developmental stage. Additionally, genes encoding E2A and bHLH-zip proteins are not selectively expressed in pre-B cells. Timing of iEκ activation is now attributed to a combination of chemokine and cytokine activity that moves pre-B cells away from an IL-7-rich milieu essential for pro-B cell differentiation and proliferation. Clark and colleagues have proposed that STAT5 activated in response to IL-7 signals binds to iEκ and suppresses its activity in pro-B cells by competing with E2A binding and/or recruitment of EZH2, a writer of repressive H3K27me3 modification (Mandal et al., 2009; Malin et al., 2010; Mandal et al., 2011). In the IL-7-poor pre-B cells niche, phospho-STAT5 binding is reduced, permitting iEκ activation (Clark et al., 2014). STAT5 is also implicated in repressing BRWD1, a transcription factor that is up-regulated in the transition to pre-B cells, which was recently shown to bind iEκ and regulate *Igk* locus contraction (Mandal et al., 2015; Mandal et al., 2024). Loss of STAT5 from iEκ also coincides with recruitment of Ikaros to this enhancer which may also regulate its developmental stage-specific activation (see below). Finally, iEκ is also associated with a MAR on its 5′ flank (Cockerill and Garrard, 1986). Though systematic transgenic studies have not been done to investigate the contribution of this MAR to iEκ function, deletion of the MAR from the endogenous locus does not affect *Igk* rearrangements or B cell development (Yi et al., 1999).

Less is known about factors that activate 3′Eκ and Ed. ChIP studies show 3′Eκ binds to PU.1, E2A, IRF and PAX5 in pro- and pre-B cells (Figure 2C) (Lin et al., 2010; Shaffer et al., 1997; Lu et al., 2003; Ochiai et al., 2012; Revilla-I-Domingo et al., 2012; Schwickert et al., 2014; Stadhouders et al., 2014). Because 3′Eκ function has only been demonstrated in combination with either iEκ or Ed, it is not clear at which developmental stage 3′Eκ is activated. Published studies show that Ed binds PU.1, E2A, and IRF4 in pro-B cells (Lin et al., 2010; Shaffer et al., 1997; Lu et al., 2003; Schwickert et al., 2014; Stadhouders et al., 2014), though it is uncertain if this binding occurs in pre-B cells as well. In contrast, Ed binds selectively to Ikaros and PAX5 in pre-B cells (Ochiai et al., 2012; Revilla-I-Domingo et al., 2012; Schwickert et al., 2014; Ferreiros-Vidal et al., 2013). How these dynamically shifting interactions contribute to Ed function is not understood. An interesting unifying feature of all three *Igk*-associated enhancers is that they bind Ikaros selectively in pre-B cells (Ribeiro de Almeida et al., 2015). Ikaros has been shown to regulate pre-B cell differentiation and proposed to induce an enhancer hub in the 3′ *Igk* locus (Hu et al., 2023). This hub promotes interactions with Vκ gene segments leading to locus contraction required for distal Vκ rearrangements. Spatial proximity of 5′ Vκs and the 3′ *Igk* region is reduced in Ikaros-deficient pre-B cells with concomitant reduction in Vκ gene rearrangements. These observations are consistent with Ikaros playing a crucial role in timing *Igk* locus activation and recombination in pre-B cells (Hu et al., 2023). The possible interplay between BRWD1 and Ikaros, both of which induce locus contraction of *Igk*, remains to be discovered.

A global view of the chromatin structure of the *Igk* locus reveals similarities and dissimilarities with *Igh.* Like *Igh,* chromatin accessibility of all three recombination-related enhancers precedes developmental stage-specific functional activation. This is reflected in strong ATAC peaks at iEκ, 3′Eκ and Ed in pro- as well as pre-B cells (Figure 2B, left panel). However, activity as reflected by germline transcription is most prominent in pre-B cells (Figure 2B, right panel). Thus, developmental timing is strictly enforced at the level of function but not at the level of chromatin accessibility. Unlike *Igh*, however, *Igk* enhancers are not pre-marked in early hematopoiesis (Figure 2B, left panel). 3′Eκ gains accessibility first at the CLP stage whereas iEκ and Ed are most prominently accessible at pro- and pre-B cell stages (Figure 2B, left panel). The close coincidence of transcriptional activation with Ikaros binding strongly suggests that transformation of accessible but functionally inactive enhancers to a transcriptionally active state is mediated by recruitment of Ikaros to pre-marked chromatin regions.

It is interesting to note that mutation of E elements in iEκ or absence of Ikaros in pre-B cells attenuate iEκ function (Inlay et al., 2004; Hu et al., 2023). E2A binds to iEκ in both pro- and pre-B cells, suggesting that pre-marking of iEκ in pro-B cells may be mediated by this protein. Furthermore, a two-fold increase of E2A transcripts was observed in pre-B cells compared to pro-B cells (ImmGen) (Heng et al., 2008), indicating a greater abundance of E2A at iEκ. The mechanism of Ikaros recruitment to iEκ could be via direct interactions with E2A or to the altered chromatin state induced by E2A. Chromatin structure analyses of E-mutated iEκ in pro-B cells and point mutation of Ikaros binding sites will help to disentangle functions of these proteins at iEκ. It is also interesting that the spectrum of transcription factors that bind to 3′Eκ is very similar to that at iEκ. Yet, 3′Eκ does not effectively compensate for genetic deletion of iEκ. The basis for this distinction is not clear but may relate to its location beyond Cκ and associated inefficiency in inducing a recombination center near the Jκ gene segments.

### Comparing stage-specific activity of Eμ and iEκ

In summary, multiple enhancers regulate developmental stage-specific transcription and rearrangements of *Igh* and *Igk* loci. Of these, Eμ and iEκ enhancers appear to be the most important because deletion of either element alone substantially impairs activation of the associated locus. A comparison of the two enhancers highlights several features:

1) Chromatin accessibility and transcriptional activation are temporally distinct for both enhancers. Accessibility at Eμ is evident throughout early hematopoiesis, whereas iEκ gains most accessibility in B lineage committed cells. Yet, transcriptional activation occurs precisely at pro- and pre-B stages, respectively (Figures 1C, 2B).
2) Both enhancers bind many of the same transcription factors, such as PU.1, IRF proteins, E2A and OCT proteins. Though these factors are important for B cell development they are unlikely to direct stage-specific enhancer activation. However, factors specific for each enhancer, such as YY1, RUNX, ETS for Eμ and Ikaros, BRWD1 and FOXO1 for iEκ are expressed in other tissues, suggesting more complex mechanisms at play than simple DNA binding. It also remains entirely possible that additional, currently unidentified, DNA binding proteins confer stage-specific enhancer activation.
3) Both enhancers are functionally inactive in the T lineage (there is residual activity of Eμ in DP cells as discussed above), despite binding transcription factors that are largely expressed in both B and T lineages. PU.1 is the exception to this rule. Its low-level expression is necessary for B cell differentiation and its extinction is essential for T cell differentiation. Further studies will be needed to understand mechanisms by which the same transcription factors activate enhancers selectively in one or the other lineage.

## Enhancers that regulate T cell receptor gene rearrangements

### Eβ control of Tcrb expression

Multipotential cells that migrate to the thymus undergo sequential rearrangements of *Tcrb* and *Tcra* genes. Developmental stages in the thymus are defined based on the expression of CD4 and CD8 cell surface proteins. The earliest stages lack both and are referred to as double negative (DN) cells (Figure 1A). DN cells are further divided into DN1, 2a/b, 3a/b and 4 (Krangel, 2009; Rodriguez-Caparros et al., 2020; Hosokawa and Rothenberg, 2021) subsets. DN1 cells are a mixed population that include multipotential cells. Commitment to T cell differentiation program is imposed in DN2 subsets by the combined action of transcription factors TCF1, BCl11b and GATA3 (Rothenberg and Taghon, 2005; Hosokawa and Rothenberg, 2021). *Tcrb* rearrangements initiate in DN2 cells and are completed by the DN3 stage. Only cells expressing TCRβ protein differentiate to CD4^+^CD8^+^ double positive (DP) stage via the intermediate DN4 stage. *Tcra* rearrangements occur in DP cells leading to generation of T cell receptor-expressing progenitor cells.

Organization of the germline *Tcrb* locus is shown in Figure 3A. The *Tcrb* locus spans approximately 0.65 Mb of mouse chromosome 6. The 5′ part contains 33 Vβ gene segments (*Trbv*) of which 21 are functional. Multiple trypsinogen genes are located between Vβ and Dβ genes. The 3′ end contains two Dβ-Jβ-Cβ clusters. Each cluster has one Dβ gene segment (Dβ1 or Dβ2), six Jβ gene segments and exons encoding the constant parts of TCRβ chains (Cβ1 or Cβ2). One Vβ gene segment, Vβ31, is located beyond the Dβ-Jβ-Cβ clusters. Despite being organized differently from *Igh*, V(D)J recombination proceeds similarly at both loci. Dβs rearranges first in DN2 cells followed by Vβ rearrangements to DJβ junctions in DN3 cells (Krangel, 2009). Three regulatory sequences control *Tcrb* rearrangements. Eβ, an enhancer located between Cβ2 and Vβ31, is essential for *Tcrb* recombination. Its deletion abrogates all *Tcrb* rearrangements (Bories et al., 1996; Bouvier et al., 1996). Absence of Eβ leads to loss of activating histone modifications (H3ac and H3K4me2) in a 25 kb region extending to a boundary element located 5′ of Dβ (Majumder et al., 2015; Mathieu et al., 2000; Carabana et al., 2011). Coordinately, this region gains repressive histone modifications, H3K9me2 and H3K27me3, on Eβ-deleted alleles (Majumder et al., 2015; Carabana et al., 2011). One side of Eβ contains a MAR, however, its deletion from the locus does not affect *Tcrb* transcription in thymocytes (Chattopadhyay et al., 1998).

**FIGURE 3:**
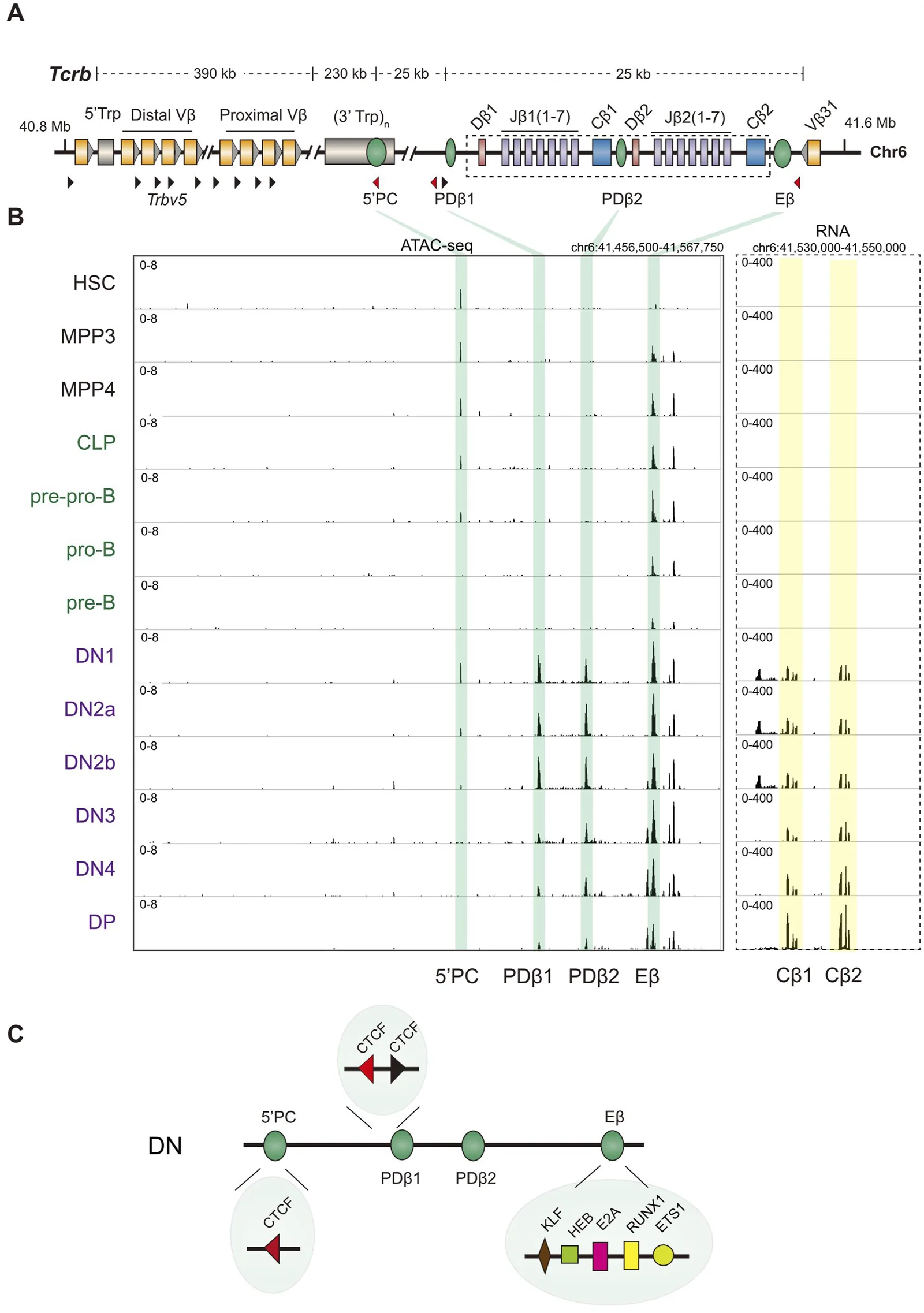
*Tcrb* locus regulatory sequences and transcription during T cell development **(A)** Schematic representation of the 0.65 Mb mouse T cell receptor β (*Tcrb*) locus, located on chromosome 6 (coordinates shown are mm10). The variable (Vβ), diversity (Dβ1 and Dβ2), joining (Jβ1 and Jβ2) gene segments are depicted as yellow, pink and purple boxes. Blue boxes represent constant region (Cβ) exons. Trypsinogen genes located between Vβ and Dβ genes are indicated as grey rectangles. Grey triangles adjacent to the Vβ gene segments indicate recombinase signal sequences (RSS) required for gene rearrangements. RSS adjacent to diversity and joining segments are not shown. Cis-regulatory elements, including enhancers (Eβ, PDβ), 5′PC CTCF site are represented as green ovals. CTCF binding sites are indicated by black and red triangles, marking opposite orientations. The region highlighted by a dashed box is expanded in the right panel of part B to display RNA-seq data. **(B)** ATAC-seq data (left panel) and RNA-seq (right panel) profiles of the 3′ part of the *Tcrb* locus during hematopoiesis obtained from the Immunological Genome project (Yoshida et al., 2019). ATAC-seq patterns covering known cis regulatory sequences are shown as identified below the tracks. For RNA-seq the pattern of the locus in the dashed box in part **(A)** is shown. **(C)** Transcription factors that binds to previously identified regulatory sequences in the 3′ *Tcrb* domain in DN cells are shown. The summary combines *in vitro* protein binding studies and *in vivo* analysis by chromatin immunoprecipitation assays (ChIP) (Majumder et al., 2015; Spicuglia et al., 2002; Zhao et al., 2017; Loguercio et al., 2018), and does not represent the numbers of each protein binding site.

PDβ1 and PDβ2 are promoters located 5′ of the respective Dβ gene segments. Deletion of PDβ1 promoter attenuates recombination of the nearest Dβ1 gene segment (Whitehurst et al., 1999; Whitehurst et al., 2000). PDβ1 lies within the domain of influence of Eβ, and its histone modification state is altered on Eβ-deleted *Tcrb* alleles (Spicuglia et al., 2002). The effects of mutating or deleting PDβ2 alone have not been investigated. 5′PC, located within the trypsinogen cluster, is a CTCF binding site. Deletion of a large genomic region between Dβ2-Jβ2 and *Trbv*5 gene segments that includes 5′PC permits rearrangement of the remaining *Trbv*5 to Dβ2. The authors concluded that 5′PC plays a regulatory role in ordered assembly of *Tcrb* genes (Senoo et al., 2003).

ATAC profile of the 3′ end of *Tcrb* shows that Eβ is accessible in bone marrow progenitors as noted above for Eµ (Figure 3B, left panel). Accessibility of 5′PC in HSC and all developmental stages leading to B and T lymphocytes probably reflects lineage non-specific binding of CTCF. Accessibility at a region near the Vβ31 promoter parallels the pattern of Eβ (Figure 3B, left panel). This region also contains a CTCF binding site which, unlike 5′PC, may require Eβ accessibility for CTCF binding. Coincident with T cell precursors reaching the thymus (DN1 cells), PDβ1 and PDβ2 become accessible. We surmise this represents Eβ activation and Eβ-dependent activation of Dβ-associated promoters. Accordingly, non-coding transcription of *Tcrb* alleles is also first evident in DN1 cells (Figure 3B, right panel). This is reminiscent of the distinction between enhancer accessibility and enhancer activation observed with Eµ. Thereafter, PDβ1 and PDβ2 remain accessible in DN2 cells as *Tcrb* rearrangements proceed. Loss of ATAC accessibility of these regions in DN3 cells likely represents their loss from the genome by Vβ recombination. Eβ accessibility and activity is maintained in further differentiated T cells, but not in other mature hemopoietic lineages such as B cells and myeloid cells (Figure 3B, left panel). Taken together, these observations are consistent with developmental timing of *Tcrb* transcription and rearrangements being determined by Eβ activation.

### Mechanisms of Eβ activation

The most prominent transcription factor motifs identified within Eβ are two composite ETS/RUNX binding sites (Figure 3C) (Hollenhorst et al., 2009). Targeted mutation of both RUNX sites abolishes enhancer activity and blocks T cell development as seen with Eβ deletion (Majumder et al., 2015). Oltz and colleagues dissected the requirements for ETS and RUNX proteins using mutated enhancers in which RUNX binding sites were replaced with GAL4 binding sites. Recruitment of RUNX1/GAL4 fusion proteins to the mutated enhancer activated Eβ-like function with regard to PDβ1 and PDβ2 transcription even in the absence of the adjacent ETS binding sites (Zhao et al., 2017). The ETS family member proposed to work at Eβ is ETS1, a gene whose expression is increased in T cell progenitors undergoing *Tcrb* rearrangements (Rodriguez-Caparros et al., 2020; Rothenberg et al., 2008; Cauchy et al., 2016). ETS1 and RUNX proteins bind cooperatively to ETS/RUNX composite motifs via neutralization of autoinhibitory domains in each factor leading to the following model for Eβ activation by these factors (Wotton et al., 1994; Kim et al., 1999; Goetz et al., 2000; Gu et al., 2000). RUNX1 is expressed throughout hematopoiesis, however it does not bind and activate Eβ until ETS1 levels rise in DN2 cells close to T lineage commitment (Rothenberg et al., 2008). Cooperative RUNX1-ETS1 binding to Eβ permits recruitment of co-activators such as CBP/p300 to establish transcriptional competence (Hollenhorst et al., 2009; Yang et al., 1998).

Several questions remain to be addressed. First, it has not been established whether the two ETS/RUNX motifs are sufficient for Eβ activity. Other transcription factors, such as E2A and the related bHLH protein HEB, have been shown to bind Eβ (Spicuglia et al., 2002) (Figure 3C), however their functional significance in the context of the *Tcrb* locus has not been explored. Second, it is not clear what makes Eβ T lineage specific because ETS and RUNX family proteins are widely expressed in hematopoietic cells. One possibility is that negative regulatory elements within Eβ suppress its activity in the wrong lineage. However, such elements have not been identified. Third, what factors make Eβ ATAC sensitive in bone marrow progenitors? Amongst key Eβ binding proteins identified to date the obvious candidate is RUNX1 (or a related RUNX family member). However, if RUNX1 proteins can bind and increase chromatin accessibility, then what prevents them from activating transcription as shown by the GAL4 fusion recruitment studies? Perhaps RUNX proteins bind with other (non-ETS) proteins in uncommitted progenitors in a configuration that precludes transcriptional coactivator recruitment. ETS1 (or other functionally similar ETS proteins) may replace these factors in DN1/2 cells to cooperatively activate Eβ with pre-bound RUNX proteins. Alternatively, progenitor accessibility may be mediated by currently unknown Eβ binding proteins. We expect that additional functional Eβ binding proteins will be identified.

### Eα control of Tcra expression

Productive rearrangement of *Tcrb* alleles in DN cells leads to proliferation and differentiation of TCRβ chain-expressing T cell precursors to the CD4^+^CD8^+^ (double positive, DP) stage (Figure 1A). *Tcra* gene rearrangements take place in DP cells, poising these cells to express the complete αβ T cell receptor. Following additional selection events in the thymus, CD4^+^ and CD8^+^ single positive cells capable of mounting immune responses emerge. *Tcra* genes arise from a single recombination event between one of ∼80 Vα gene segments (*Trav*) and one of approximately 60 Jα gene segments (*Traj*) located close to exons that encode the constant part (Cα, *Trac*) of TCRα chains (Figure 4A). A 1.7 Mb region of mouse chromosome 14 houses the *Tcra* locus with interspersed gene segments that will recombine to generate TCRδ chains of the γδ T cell receptor (Figure 4A). *Tcrd* diversity (Dδ, *Trdd*), joining (Jδ, *Trdj*) and a few variable (Vδ, *Trdv*) gene segments, along with constant parts of the TCRδ chains (Cδ, *Trdc*), are embedded between *Traj* and most of the *Tcra* variable gene segments (*Trav*) (Figure 4A). Despite being located within the *Tcra* locus, *Tcrd* gene rearrangements occur in DN cells guided by the Eδ enhancer located near *Trdc*. Loss of Eδ selectively abolishes *Tcrd* rearrangements without affecting *Tcra* rearrangements (Monroe et al., 1999). Though Eδ will not be further discussed in this review it is interesting to note that its activity, as measured by *Trdc* transcription, is restricted precisely to DN cells where *Tcrd* rearrangements occur (Figure 4B).

**FIGURE 4:**
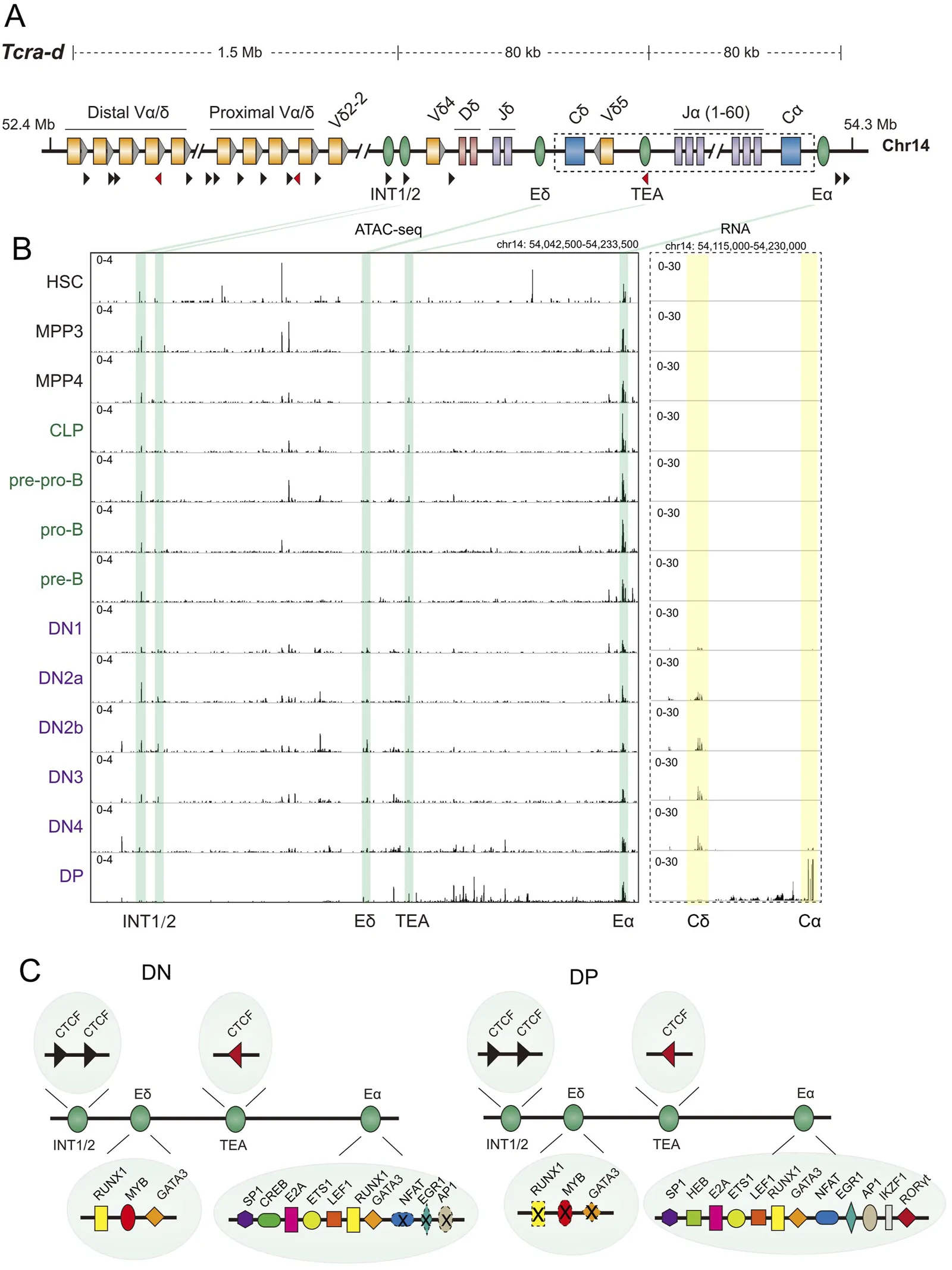
*Tcra-d* locus regulatory sequences and transcription during T cell development. **(A)** Schematic representation of the mouse T cell receptor α/δ (*Tcra-d*) locus, spanning approximately 1.6 Mb on chromosome 14 (coordinates are in mm10). Variable (Vα/δ (*Trav* and *Trdv*)), diversity (Dδ (*Trdd*)) and joining (Jα/δ (*Traj* and *Trdj*)) gene segments are shown as yellow, pink and purple boxes. Blue boxes represent constant region (Cα/δ (*Trac* and *Trdc*)) exons. Grey triangles adjacent to the Vα/δ gene segments indicate the RSSs. Cis-regulatory elements, including enhancers (Eδ and Eα), intergenic CBEs 1 and 2 (INT1 and INT2), and T early alpha (TEA) promoter, are represented as green ovals. CTCF binding sites and orientations are shown by black and red triangles. The region highlighted by the dashed box is expanded in the right panel of part B to display RNA-seq data. **(B)** ATAC-seq (left panel) and RNA-seq (right panel) profiles of the 3′ region of the *Tcra-d* locus during hematopoiesis obtained from the Immunological Genome project (Yoshida et al., 2019). ATAC-seq patterns covering known cis regulatory sequences are shown as identified below the tracks. For RNA-seq, the pattern of the locus in the dashed box in part **(A)** is shown. **(C)** Transcription factors that bind to previously identified in the 3′ *Tcra-d* domain in double negative (DN) and double positive (DP) cells are shown (Schwickert et al., 2014; Hernandez-Munain, 2015; del Blanco et al., 2012; Naik et al., 2024; Loguercio et al., 2018; Wei et al., 2011; Mihai et al., 2023). The summary combines *in vitro* protein binding studies and *in vivo* analysis by chromatin immunoprecipitation (ChIP) and does not represent the numbers of each protein binding site. Sites depicted with dashed lines and a cross identify unoccupied sites at the specified developmental stage.

The Eα enhancer located 3′ of Cα is essential for *Tcra* rearrangements. This enhancer is marked by an ATAC peak at all hematopoietic stages, including HSC (Figure 4B, left panel). Indeed, accessibility appears stronger in B cell precursors than in T cell precursors. Yet, its activity, as inferred by Cα RNA, is exquisitely specific to the developmental stage (DP cells) at which *Tcra* genes rearrange. Eα and approximately 80 kb of 5′ sequence that include Cα exons and all Jα gene segments gain histone H3 acetylation during transition to DP cells (McMurry and Krangel, 2000), closely correlating with transcriptional activation of *Tcra*. This domain of active histone modifications is lost in DP cells from mice that lack Eα, showing that the enhancer regulates long-range chromatin state that correlates with gene recombination (McMurry and Krangel, 2000). A MAR has not been identified near Eα (Scheuermann and Garrard, 1999). The ATAC pattern is reminiscent of those at *Igh* and *Tcrb* loci and distinct from that at *Igk*, insofar as enhancers in *Igk* are not accessible in early bone marrow precursors. Thus, locus activating enhancers at three out of four major antigen receptor loci are pre-marked early in hematopoiesis but activated in the appropriate lineage at the correct developmental stage.

In addition to Eα, several CTCF-binding regulatory elements modulate *Tcra* rearrangements. The T early alpha (TEA) promoter guides use of Jα gene segments. Its deletion results in reduced rearrangements of several 5′ Jα segments closest to TEA, but no effect on Jα segments located further 3’ (Villey et al., 1996). Intergenic CBEs 1 and 2 (INT1 and INT2) separate the large genomic region that contains *Trav* and *Trdv* gene segments from the rest of the *Tcra-d* locus. Orientation of CTCF binding sites in each element define an 80 kb domain that extends till the CTCF binding site in TEA (Chen et al., 2015). This domain, containing Dδ, Jδ and Cδ under control of Eδ, is thereby effectively segregated from the rest of the locus. Double deletion of INT1 and INT2 alters the *Tcra* repertoire and leads to defective γδ T cell development. The two elements are partially redundant as mutation of INT2 alone has minor effects (Chen et al., 2015).

### Mechanisms of Eα activation

Many transcription factors bind Eα both in DN cells (where it is inactive) and in DP cells (where it is active, Figure 4C) (Hernandez-Munain, 2015). Amongst these are the first examples of T lineage-restricted factors such as LEF1/TCF1 and GATA3. These factors are expressed concomitant with T cell commitment in DN2 cells simultaneously with *Tcrb* activation by Eβ. However, their binding (along with other proteins) is apparently insufficient to activate Eα. By contrast, three factors that are widely associated with cell activation and pre-T cell receptor (pre-TCR) signaling, NFAT, EGR and AP1, bind selectively to Eα in late DN4 cells and DP cells (King et al., 1999; Aifantis et al., 2001; Carter et al., 2007; del Blanco et al., 2012). Hernández-Munain and colleagues proposed that constitutive but lymphoid-restricted transcription factors, such as E2A and ETS1, pre-mark Eα before its activation. The pre-marked enhancer recruits additional factors induced by pre-TCR signaling, as well as the CREB-binding protein/p300 coactivators, to fully activate Eα (del Blanco et al., 2012).

RORγt and Ikaros are two other proteins that were recently shown to bind to Eα in DP cells by ChIP experiments (Schwickert et al., 2014; Naik et al., 2024); whether they bind to Eα at earlier stages has not yet been explored. RORγt is important for lifetime of DP cells which, in turn, impacts the Vα repertoire (Sun et al., 2000; Guo et al., 2002). However, a direct role for RORγt in regulating recombination via Eα activity has not been demonstrated. It is noteworthy that Ikaros binds to both late-activated antigen receptor gene enhancers (iEκ and Eα) at the appropriate developmental stage. Because Ikaros deficiency perturbs T cell development at the earliest stages (Georgopoulos, 2002), it is not known to what extent Ikaros binding to Eα is required for enhancer function in DP cells. Point mutational analyses of Eα will be needed to sort through transcription factor dynamics and functions in developing T cells.

### Comparing stage-specific activity of Eβ and Eα

Unlike *Igh* and *Igk* where multiple enhancers guide developmental stage-specific locus activation, *Tcrb* and *Tcra* loci each rely on only one (known) enhancer to initiate developmentally appropriate transcription and rearrangements. A comparison of Eβ and Eα is therefore pertinent for identifying mechanisms that guide T cell stage-specific gene expression. Additionally, a comparison of BCR- and TCR-associated enhancers provides a perspective into lineage-restricted gene expression. Several interesting features can be highlighted:

1) Both Eβ and Eα are ATAC sensitive from early hematopoietic stages. Thus, chromatin accessibility can be dissociated from transcriptional activation at all antigen receptor gene enhancers. This raises interesting questions regarding the role of pioneer factors in lineage- and stage-specific activation of antigen receptor gene enhancers. It is possible that these enhancers are atypical because of their role in recombination regulation beyond classical transcription activation. It is also interesting to note that both Eβ and Eα remain accessible in B lineage precursors, whereas both Eμ and iEκ are mostly inaccessible in T cell precursors.
2) Organization of Eβ, controlled by RUNX and ETS proteins, appears much simpler than that of Eα. However, both these transcription factors and (and many others) bind Eα in DN cells without apparently activating transcription. One possibility is that the numbers of RUNX/ETS motifs matter, to confer activity (of Eβ) or inactivity (of Eα) in DN cells. Additional factors, such as bHLH proteins, also bind to both Eβ and Eα in DN cells, leaving Eα inactivity in DN cells a mystery.
3) Both iEκ and Eα are transcriptionally activated as pre-BCR- or pre-TCR-selected progenitors complete a proliferative burst and regain quiescence as pre-B or DP cells, respectively. Their mechanisms of activation, however, appear to be quite different. iEκ activation has been attributed to pre-BCR-dependent reduced sensitivity to IL-7, whereas Eα activation coincides with recruitment of activation-induced transcription factors. Loss of IL-7 signaling is also associated with DN to DP transition of T cell progenitors and, conversely, activation-induced transcription factors are likely to be in play during pro- to pre-B transition of B cell progenitors. It is intriguing that apparently different strategies are adopted in the two lineages to accomplish the same end.

### Organization and transcription factor utilization at Eμ and Eβ

It is interesting to examine the mechanisms by which Eμ and Eβ are transcriptionally activated coincident with B or T lineage commitment from multipotential progenitors. Both Eμ and Eβ contain multiple binding sites for bHLH, RUNX and ETS proteins, yet each largely activates transcription and recombination in distinct lineages. One possibility is that this is due to different organization of binding sites within each enhancer. For example, only one of the four RUNX binding sites in Eμ has the configuration of ETS/RUNX composite elements that dominate Eβ. ETS binding sites in Eμ are also distinct in both family member selectivity (Eμ has two PU.1 binding sites whose sequence specificities are different from that of ETS1 and related factors) and distribution across the enhancer. Both Eμ and Eβ are also pre-marked by chromatin accessibility much earlier in hematopoiesis than their functions are manifest (Figures 1C, 3B). We hypothesize that pre-marking identifies genomic sites at which functional factors will be recruited in the appropriate lineage and at the appropriate developmental stage. It will be interesting to identify other such regulatory sites that are pre-marked early in hematopoiesis for later functional activation to understand the underlying regulatory logic. It will also be interesting to compare such regulatory sequences to those at which accessibility and functional activation coincide. In the context of pioneer factors these observations suggest that factors that drive Eμ and Eβ activity may not be capable of pioneering correctly.

## Concluding remarks

In this review we have summarized features of developmental regulation of antigen receptor loci from the perspective of enhancers associated with immunoglobulin (Ig) and T cell receptor genes. Though analyses of these enhancers led to identification of some of the key transcription factors required for B and T cell development, it is apparent that mechanisms by which they direct lineage- and developmental stage-specific activation remain poorly understood. It is also apparent that ‘simple’ identification of transcription factors binding sites within enhancers will not suffice to explain how overlapping sets of factors yield developmentally precise gene activation. The concept of combinatorial control was evoked to explain this but how it is imposed remains a challenge for the future. It is also intriguing that enhancer accessibility is distinct from enhancer activity, perhaps explaining in part their complex organization. To what extent this is true of other tissue-specific control elements remains unclear, as does the underlying reason. Finally, MARs are associated with three out of the four antigen receptor enhancers that control recombination. Functions of these enigmatic regulatory sequences remain to be elucidated.
